# COVID-19: Sleep, Circadian Rhythms and Immunity – Repurposing Drugs and Chronotherapeutics for SARS-CoV-2

**DOI:** 10.3389/fnins.2021.674204

**Published:** 2021-06-18

**Authors:** Allan Giri, Ashokkumar Srinivasan, Isaac Kirubakaran Sundar

**Affiliations:** Division of Pulmonary, Critical Care and Sleep Medicine, Department of Internal Medicine, University of Kansas Medical Center, Kansas City, KS, United States

**Keywords:** SARS-CoV-2, COVID-19, sleep, circadian rhythms, immunity, Chronotherapy

## Abstract

The severe acute respiratory syndrome coronavirus 2 (SARS-CoV-2) pandemic has affected nearly 28 million people in the United States and has caused more than five hundred thousand deaths as of February 21, 2021. As the novel coronavirus continues to take its toll in the United States and all across the globe, particularly among the elderly (>65 years), clinicians and translational researchers are taking a closer look at the nexus of sleep, circadian rhythms and immunity that may contribute toward a more severe coronavirus disease-19 (COVID-19). SARS-CoV-2-induced multi-organ failure affects both central and peripheral organs, causing increased mortality in the elderly. However, whether differences in sleep, circadian rhythms, and immunity between older and younger individuals contribute to the age-related differences in systemic dysregulation of target organs observed in SARS-CoV-2 infection remain largely unknown. Current literature demonstrates the emerging role of sleep, circadian rhythms, and immunity in the development of chronic pulmonary diseases and respiratory infections in human and mouse models. The exact mechanism underlying acute respiratory distress syndrome (ARDS) and other cardiopulmonary complications in elderly patients in combination with associated comorbidities remain unclear. Nevertheless, understanding the critical role of sleep, circadian clock dysfunction in target organs, and immune status of patients with SARS-CoV-2 may provide novel insights into possible therapies. Chronotherapy is an emerging concept that is gaining attention in sleep medicine. Accumulating evidence suggests that nearly half of all physiological functions follow a strict daily rhythm. However, healthcare professionals rarely take implementing timed-administration of drugs into consideration. In this review, we summarize recent findings directly relating to the contributing roles of sleep, circadian rhythms and immune response in modulating infectious disease processes, and integrate chronotherapy in the discussion of the potential drugs that can be repurposed to improve the treatment and management of COVID-19.

## Introduction

The novel coronavirus virus, SARS-CoV-2, has affected the lives of people in almost every corner of the globe and imposed threats and several challenges on the healthcare front. Since the emergence of this novel virus in Wuhan, China in late November 2019, 3.02 million people have lost their lives worldwide as of April 20, 2021 (Johns Hopkins University and Medicine, 2020). As the death toll continues to rise in the United States and all across the globe, clinicians and translational researchers are taking a closer look at the molecular cross-talk between sleep, circadian rhythms and immunity that may play an essential role during the COVID-19 pandemic. SARS-CoV-2-induced multi-organ failure affects both central and peripheral organs, causing increased mortality in the elderly (Garg et al., 2020). However, whether differences in sleep, circadian rhythms and immunity between older and younger individuals contribute to the age-related differences in systemic dysregulation of target organs observed in SARS-CoV-2 infection is still being investigated. Accumulating evidence from the literature demonstrates the emerging role of sleep, circadian rhythms and immunity in the development of chronic pulmonary diseases and respiratory infections in human and mouse models (Sundar et al., 2015c). The exact mechanism underlying acute respiratory distress syndrome (ARDS) and other cardiopulmonary complications in elderly patients in combination with associated comorbidities remains unclear, yet understanding the nexus of sleep, circadian clock dysfunction in target organs and immune status of patients with SARS-CoV-2 may provide novel insights into possible therapies.

The circadian timing system is a lot more complex process than a simple internal machinery that regulates our sleep-wake cycle. It has been established categorically that the circadian rhythms can regulate our innate and adaptive immune response, modulate viral replication within the host cells, coordinate physiological processes, and often determine the severity of many illnesses (Zhuang et al., 2017; Haspel et al., 2020). Prior evidence indicates that if the circadian system is disrupted, the vulnerability of host cells toward influenza and herpes virus increases (Edgar et al., 2016; Mazzoccoli et al., 2020; Zhuang et al., 2021). It may seem counterintuitive but the truth is occurrences of circadian disruption and disturbances in sleep had been more frequent during the pandemic despite social confinement (Morin et al., 2020). For maintaining good physical health and ensuring optimum functionality of the immune system, adequate amount of good quality sleep is mandatory. Sleep promotes mental and emotional well-being which is key for driving away anxiety, depression and stress (Medic et al., 2017). The COVID-19 pandemic has triggered anxiety, stress and fright among citizens of the United States and globally, which indirectly disrupts the circadian rhythms and increases the disease severity in COVID-19 patients (Huang B. et al., 2020).

Chronotherapy is an emerging concept that is being increasingly discussed in sleep medicine. Through this, medication is administered to patients in sync with their circadian rhythms. Chronotherapy can potentially reduce the side effects of the administered drugs while optimizing the therapeutic impact (Selfridge et al., 2016; Cederroth et al., 2019; Ruben et al., 2019). Additionally, it can also achieve the same efficacy at a lower dose (Matsuzawa et al., 2018). COVID-19 patients might tremendously benefit from chronotherapy. For instance, a recent report indicated that statins significantly reduce COVID-19 severity by reducing cholesterol, which is used by the SARS-CoV-2 to infect the lung epithelial cells, making hyperlipidemia a risk factor for mortality among COVID-19 patients (Aung et al., 2020). Strong evidence exists that cholesterol synthesis peaks during the nighttime hours between 12 and 6 am, and that administering the same dose of statin in the evening or nighttime markedly reduced cholesterol levels in patients when compared to the same dose given in the morning (Izquierdo-Palomares et al., 2016). In this article, we will summarize the critical role of sleep and circadian rhythms in health and disease and suggest ways to reset the circadian timing system. Additionally, we discuss some major classes of drugs that can be repurposed for the treatment and management of COVID-19 in the light of chronotherapy and highlight some of the knowledge gaps in this domain for the scope of future research.

## Sleep and Circadian Rhythms

Sleep is a normal physiological and behavioral state of the body, described as reversible unconsciousness with minimal physical movement and non-responsiveness to external stimuli. Evolutionarily, all animal species, including vertebrates (e.g., mammals, fish, and birds) and invertebrates (e.g., insects, roundworms, and jellyfish), exhibit some characteristics of sleep or sleep-like states (Keene and Duboue, 2018). Sleep and circadian rhythms are complex and tightly interconnected mechanisms. In humans, the central pacemaker, or “clock,” resides in the suprachiasmatic nucleus (SCN) of the hypothalamus in the brain. The SCN controls the periodic release of melatonin, a hormone that induces sleep in humans. It primarily receives stimulus in the form of light via the optic nerve, which is responsible for relaying the stimulus from the eye to the brain. Melatonin secretion is inhibited by light while stimulated by darkness. At night when light is scarce, the SCN stimulates the release of melatonin from the pineal gland and thus inducing sleep. On the other hand, melatonin feeds back to the SCN to decrease the neuronal firing of the SCN and the cycle resets (Doghramji, 2007). Additionally, light is also able to entrain the clock at the level of the SCN, which subsequently entrains the cell-autonomous clock that resides in the peripheral cells and tissues thus impart the circadian rhythms of gene expression in them (Haspel et al., 2020). A comprehensive review and perspective on the basic concepts and molecular mechanism that involve the circadian clock in lung pathophysiology of chronic airway disease has been previously reviewed (Sundar et al., 2015c,b).

**FIGURE 1:**
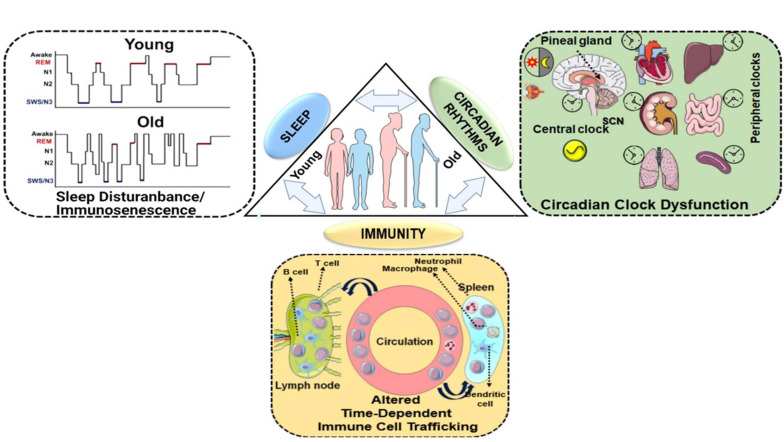
Homeostasis between sleep, circadian rhythms and immunity: a bidirectional relationship. Sleep and circadian rhythms are recognized as important intertwined regulators of the immune system. Disruption of either of the processes can lead to altered immune response, functional immunocompromise and inflammation. The lack of sleep, shorter sleep duration, or sleep disturbances greatly increases our susceptibility to infections as a result of an altered immune response. The reduced mitogenic proliferation of lymphocytes decreased HLA-DR expression and variations among the T lymphocytes, have been observed in sleep-deprived individuals. Conversely, the immune response has also been shown to alter sleep patterns. Immune response to a pathogen is accompanied by the release of cytokines and interleukins, which depending on the magnitude, can either facilitate or cause sleep disruption. Sleep enhancement is usually assumed to be a defense mechanism for the host. Similarly, sleep and circadian rhythms are coordinated as well. Light plays the most important role in providing an environmental cue to the circadian rhythms. When light hits the retinal ganglion cells, the SCN switches off the melatonin secretion and, in turn, diminishes sleep. At nighttime, when lights are off, the SCN trigger the release of melatonin, which facilitates sleep. Melatonin levels decline as people age, the old generation experience a shorter duration of slow-wave sleep (SWS) that interferes with the function of circadian rhythms as shown in the schematic. Chronic exposure to light at nighttime, consuming caffeine, alcohol disrupt the sleep schedule and thus affect the circadian clock. Given, the wide range of physiological functions that are under circadian control, care must be taken to maintain the normal homeostasis. This schematic was prepared from SMART (Servier Medical Art), licensed under a Creative Common Attribution 3.0 Generic License. http://smart.servier.com/.

Disruption of circadian rhythms can occur due to changes in normal daily activities such as shift work, jet lag, unusual photoperiods (e.g., polar regions), or possibly due to sleep disorders (Blask, 2009; Mattis and Sehgal, 2016). Additionally, aging plays a critical role in causing sleep-related disorders among the elderly population. It has been reported that the ability of the circadian clock to adapt to changes in the light-dark schedule, either due to jet lag, shift work, or other changes to the light-dark exposure regime reduces with increasing age (Monk et al., 2000). Evidence from the literature suggests that aged individuals demonstrate fragmented sleep patterns compared to younger individuals and that the duration of slow-wave sleep (SWS: deep sleep) decreases with increasing age (Mattis and Sehgal, 2016). Age-related neurodegenerative diseases such as Alzheimer’s, Parkinson’s, and Huntington’s show signs of circadian disruption, which is indicative of older individuals having an increased susceptibility to circadian dysfunction-associated clinical ailments including cancer risk (Blask, 2009; Abbott and Videnovic, 2016; Mattis and Sehgal, 2016). Generally, sleep helps maintain normal physiological processes such as brain development, plasticity, memory, learning and immunity (Bollinger et al., 2010; Abel et al., 2013; Figure 1). Circadian disruptions have been documented to have detrimental effects on an individual. This is more prominent in shift workers who have to continuously change biological rhythms to work at different shifts, thus and have a great chance of developing metabolic diseases, viral infections, cardiovascular diseases, diabetes, obesity, cancer or fibrosis (Almeida and Malheiro, 2016; Kecklund and Axelsson, 2016). Recent evidence also shows that shift workers have a greater likelihood of testing COVID-19 positive in hospital settings compared to that of non-shift workers, and this association remained increased regardless of the time of the shift (Maidstone et al., 2020). Although exact reasons are not known, circadian misalignment has shed some light on why this might be the case as described below.

## Circadian Rhythms-Sleep-Immunity: Crosstalk in Health and Disease

Past studies have clearly demonstrated the relationship between sleep, immunity and aging (Dickstein and Moldofsky, 1999; Besedovsky et al., 2019). During the early resting period, secretion of growth hormone (GH), melatonin, prolactin and leptin are increased, resulting in immune cell activation, proliferation, differentiation and production of pro-inflammatory cytokines (e.g., interleukins [IL-1 and IL-2], tumor necrosis factor-alpha [TNFα]) (Dickstein and Moldofsky, 1999; Esquifino et al., 2004; Hattori, 2009; Radogna et al., 2010). As mentioned previously, as individuals age, they show a higher incidence of disrupted sleep patterns, which leads to a decrease in amplitude of GH, melatonin and other hormone secretion (Kern et al., 1996; Pandi-Perumal et al., 2006). Prior report has demonstrated the effect of sleep deprivation on the immune response to influenza vaccination. Antibody titers measured 10 days post-immunization in sleep-deprived subjects were lower than half of those measured in subjects with normal sleep patterns (Spiegel et al., 2002). Another report showed the role of Gα_*s*_-coupled receptor agonists (isoproterenol, epinephrine, norepinephrine, prostaglandin E2, prostaglandin D2, and adenosine) in blocking T cell receptor (TCR)-induced β_2_-integrin activation in human Cytomegalovirus (CMV)- and Epstein-Barr virus (EBV)-specific T cells in a dose-dependent manner (Dimitrov et al., 2019). Interestingly, sleep suppresses Gα_*s*_-coupled receptor signaling, but increased β_2_-integrin activation in CMV- and EBV-specific CD8^+^ T cell subsets (specifically in the early and intermediate differentiation states, but not late) that occur early in the morning (between 2 and 6 am; dark phase) (Dimitrov et al., 2019). These findings highlight the immune-enhancing effects of sleep, wherein it regulates normal circadian rhythms to provide an optimal immune response (e.g., via formation of immunological synapses by T cell adhesion to antigen-presenting cells or target cells) and resistance to infectious challenge. Ongoing and future clinical trials for COVID-19 vaccination and therapy in humans should consider these caveats, quality of sleep index and combining the use of specific Gα_*s*_-coupled receptors agonist such as catecholamines, PGs and adenosine that may constitute immune checkpoint inhibitors, when treating SARS-CoV-2 patients with comorbid chronic underlying pathologies.

Additionally, the influx of circulating immune cells (neutrophils, natural killer [NK] cells, monocytes and B cells) occurs during prolonged wakefulness, which is then reduced by recovery sleep, eluding to the crucial role of sleep in regulating immune cell trafficking (Ingram et al., 2015; Besedovsky et al., 2019). All the immune cell types including the lymphoid organs retain the functional clock (Labrecque and Cermakian, 2015; Scheiermann et al., 2018). Documented evidence suggests that both humans and mice display daily oscillations in levels of circulating leukocytes, and that the circadian rhythms control leukocyte trafficking-mediated immune function (Scheiermann et al., 2012; Zhao et al., 2017; He et al., 2018). Aging affects the normal physiology and hormonal responses, including circadian rhythms in SCN, the central player that coordinates sleep and sleep-associated age-related disorders. Hence, disruption of circadian rhythms can impose a weaker immune response because of decreased sleep duration or age-related reduction in the period of sleep. A self-reported survey by the National Health and Nutrition Examination Surveys (NHANES; 2005-2012) that enrolled 22,726 participants showed a direct association between sleep duration (short duration of sleep) and an increased incidence of respiratory viral infections (Prather and Leung, 2016).

Lack of sufficient human and animal studies that directly relate to the role of sleep, circadian rhythms and immunity in the context of aging makes it difficult to speculate upon the role of age-related sleep disruption in the increased prevalence of respiratory bacterial and viral infections in older populations. However, epidemiological studies including human and animal experimentation have directly or indirectly indicated that sleep deprivation alters specific immune processes (Ingram et al., 2015). A few examples of this include elevated levels of systemic inflammatory mediators, increased susceptibility to viral infections, and altered adaptive immune response to influenza vaccination. Additionally, there is evidence of chronic sleep deprivation in animals causing death due to splenic atrophy and polymicrobial bacteremia possibly due to immune dysregulation (Rechtschaffen et al., 1983; Everson, 1993; Toapanta and Ross, 2009). Preclinical studies from our lab and others have shown how deletion of the circadian clock gene Bmal1 (Bmal1 KO) alters viral burden, the chance of survival, and the degree of lung inflammation and remodeling following influenza A virus (IAV) infection, albeit with altered circadian phase and amplitude (Sundar et al., 2015a; Sengupta et al., 2019). Constitutively low expression of Bmal1 results in enhanced susceptibility to Herpes simplex virus 1 and IAV infection. Additionally, genetic disruption of clock molecule Bmal1 augments viral replication in mice and cell culture models thereby demonstrating the dynamics of circadian clock-dependent host-virus interaction (Edgar et al., 2016). In a recent study, observed time of day-dependent alterations in IAV-induced lung inflammation and fatality in mice demonstrate the importance of circadian clock control in modulating immune response (Sengupta et al., 2019). WT mice infected with IAV showed temporal gating [mice infected at zeitgeber time 11 (ZT11) showed greater mortality, weight loss, clinical severity scores, and respiratory distress compared to mice infected at ZT23] while inducible Bmal1 KO mice infected with IAV showed loss of this temporal gating response (Sengupta et al., 2019). In IAV-infected mice, a higher survival rate was associated with a time-dependent increase in NK and NKT cells and a reduction of inflammatory monocytes in the lungs, indicative of the underlying circadian clock-mediated gating response (Sengupta et al., 2019).

In a recent report, circadian rhythms of druggable host factors that interact with SARS-CoV-2 proteins were analyzed using the Circadian Expression Profiles Database (CircaDB^1^). The majority of these human and mouse orthologous host proteins that are targeted by FDA-approved drugs showed a greater degree of protein-protein interaction with SARS-CoV-2 proteins, exhibit 24-h oscillation under constant conditions (Ray and Reddy, 2020). Previous report shows that glucocorticoid receptor signaling affects the circadian clock bidirectionally (Caratti et al., 2018). Studies demonstrated that therapeutic administration of glucocorticoids has been linked with increased incidence of reactivation of Hepatitis B virus (HBV) and caused poor clinical outcomes during influenza-associated ARDS (Hatano et al., 2019; Tsai et al., 2020). Melatonin on the other hand reduces inflammation by blocking NLRP3 inflammasome thereby indirectly regulating circadian rhythms and viral infection (Borrmann et al., 2021). The putative role of circadian clock role in the pathophysiology of SARS-CoV-2 infection based on prior evidence from available literature was summarized recently (Meira et al., 2020). Circadian changes in the expression of angiotensin-converting enzyme 2 (ACE2) may exist in the lungs and other peripheral organs which is not yet reported. We know that angiotensin II affects the rhythmic expression of Per2 in the SCN and heart of rats *in vivo* (Herichova et al., 2013) and circadian clock genes (Per2, Dbp, and Bmal1) in vascular smooth muscle cells *in vitro* (Nonaka et al., 2001). Hence, we speculate that altered ACE2 expression in the lung may have circadian changes that indirectly affect the renin-angiotensin system during SARS-CoV-2 infection-induced ARDS. Since circadian clock genes directly regulate functional and physiological outcomes in the lungs and cardiovascular system, it is more likely that clock-dependent immune dysregulation may be the cause for adverse pathobiology observed in SARS-CoV-2 infection which needs to be further explored.

The importance of understanding the nexus of circadian rhythms, sleep and immunity remains one of the highest priorities for translational biomedical research, which was highlighted recently in the National Institute of Health (NIH)-sponsored workshop “Sleep insufficiency, circadian misalignment and the immune response” summary report (Haspel et al., 2020). Overall, there is ample evidence for the role of the circadian clock and sleep in maintaining immune homeostasis (i.e., innate and adaptive immune response) (Pick et al., 2019; Haspel et al., 2020). Prior observations elegantly support the occurrence of time-of-day-dependent changes in leukocyte trafficking and mobilization, cytokine-mediated chemotaxis and T cell differentiation (Pick et al., 2019; Haspel et al., 2020). Understanding the role of circadian rhythms-sleep-immunity crosstalk during SARS-CoV-2-induced respiratory infection and multi-organ failure may enable us to identify novel drug targets for the treatment and management of coronavirus-induced infectious disease (Figure 1).

## Ways to Optimize Circadian Rhythms

Light is the most powerful environmental cue for the human circadian timing system. Besides the classical rod and cones photoreceptors, there is another subpopulation of retinal ganglion cells known as the intrinsically photosensitive retinal ganglion cells (ipRGCs) that express the photopigment melanopsin. These ipRGCs in the eye detect the light signal and relay the message to the SCN, the master regulator (hypothalamus) of the brain (Shuboni and Yan, 2010; Fernandez et al., 2018). The sensitivity of the photopigments on these retinal ganglion cells are more for shorter wavelengths of light like blue and green, and less for red-shifted lighting. When diurnal animals are exposed to lower intensities of blue light during the nighttime, they experience trouble falling asleep due to inhibition of sleep-inducing neurons, and subsequently, there is a significant reduction of the natural sleep hormone, melatonin. Light also stimulates the activation of the sympathetic nervous system making us more alert and conscious (Ramsey et al., 2013). Thus, sleep quality deteriorates and alertness level on a subsequent day reduces. From an evolutionary point of view, humans are diurnal animals. Chronic exposure to light at night, when humans are supposed to be asleep, has been shown to have detrimental effects on the immune system, particularly on the inflammatory response of the immune system during infection (Bedrosian et al., 2011). Many studies have found a profound association between chronic light exposure at night and the increased risk for developing certain medical conditions such as breast cancer, anhedonia (loss of interest in activities that once used to be pleasurable), depression, and elevated body mass index. The discussion of how chronic light exposure at night increases the chances of acquiring these medical conditions is beyond the scope of this review and has been summarized previously (Fonken and Nelson, 2011). In circadian biology, the synchronizing effect of light on the circadian timing system is characterized graphically on a phase-response curve (PRC). Light-induced phase shifts can be visually observed in the PRCs. In general, light stimuli late in the day or early at night generates a phase delay on the PRC while light stimuli late at night or early in the day generate a phase advance (Czeisler et al., 2005). These findings are important as it highlights how light acts as a powerful modulator for the circadian timing system and how phototherapy can be used to treat sleep phase disorders in humans. While sometimes it is hard to have a strict sleep schedule, studies have shown that maintaining a strict sleep schedule can help strengthen the circadian rhythms and facilitate better sleep quality. Frequent changes in the sleep schedule can affect the quality of sleep, decrease cognitive performance, and may increase the risk of cardiovascular events (James et al., 2017). Additionally, studies have found that irregular bedtime often leads to shorter sleep durations which can negatively impact cognition, alertness, memory and mood (Kang and Chen, 2009). Partial sleep deprivation leads to the impairment of lymphocyte proliferation, decreased HLA-DR expression, upregulation of CD14^+^, and variations among key T lymphocytes like CD4^+^ and CD8^+^, which in turn increases our susceptibility to diseases (Wilder-Smith et al., 2013). Hence, chronic exposure to light at nighttime must be avoided, which will in turn help reinforce a proper sleep schedule.

Skipping late-day naps and practicing pre-bedtime relaxation exercises may also help ensure proper bedtime. While it is true that short naps, ideally 10 to 15 min, can feel refreshing especially when a person is sleep-deprived, longer and late-day naps can actually impact the quality of nighttime sleep (Monk et al., 2001). The sleep cycle of a healthy adult is subdivided into four stages. The first two stages consisting of light, non-rapid eye movement (NREM) sleep, during which the heart and breathing rate slows down and brain activity gradually declines. The third stage comprises deep NREM sleep where brain activity, breathing and heart rates would be the lowest. The final stage is the rapid eye movement (REM) sleep where there is a surge in brain activity with a rapid movement of the eye. Typically, it is easier to wake up during the first two stages of the NREM sleep but it is difficult during the third stage. Arousal from sleep during the third stage is accompanied by grogginess and feeling of confusion. Naps later during the day usually comprise of deep sleep when there is a natural decline of our energy, and this, in turn, affects our ability to fall asleep during the night (Brooks and Lack, 2006). Surveys conducted in the United States revealed that approximately 30 to 50% of Americans have some form of insomnia (Ancoli-Israel and Roth, 1999). Stress and anxiety may be the direct or indirect consequence of this medical condition. During periods of high stress, our sympathetic nervous system is most active, increasing our heart rate and stress hormone levels. This, in turn, makes us feel more awake and alert (Valentino and Foote, 1988; Han et al., 2012). Thus, trying to fall asleep during this stress response can be challenging. Fortunately, research has shown that this stress response can be mitigated by calming the mind and relaxing the body, which can facilitate sleep naturally. This relaxation response can be activated consciously. Relaxation exercise like breathing exercises, where we can consciously control the speed and depth of our breathing; meditation, where we can consciously focus on scanning parts of our own body while letting go of any stress-inducing thoughts, light yoga, and progressive muscle relaxation, where specific muscles groups of the body can be consciously contracted and relaxed periodically (Norelli and Krepps, 2018). These simple relaxation techniques have been shown to lower the heart rate and blood pressure, create a sense of calm and reduce stress, and thus help us fall asleep faster.

Circadian rhythms can also be disrupted by an imbalance in metabolic functions, and prior studies have indicated that this can happen due to food intake at an inappropriate period or irregular eating habits (Wehrens et al., 2017). The circadian system impacts metabolic homeostasis by regulating daily physiological processes like gastrointestinal functions, absorption of nutrients, gastric acid secretion, colonic motility, appetite, and secretion of pancreatic insulin (Hoogerwerf, 2006). SCN’s neural and humoral signals coordinate the functions for driving and modulating the feedback loop of molecules in metabolic tissues such as the pancreas and liver. The circadian function can be altered by the food’s nutritional constitution, intake volume, frequency and timing. In mice, for instance, restriction to accessing food during unsuitable periods in the day like normal sleep time results in shifting the molecular clock’s phase in fat cells, adrenals, liver, and other peripheral tissues while unaltering the SCN rhythm (Damiola et al., 2000). This disrupts the coordination between the SCN and the peripheral tissues. Additionally, high-fat diets have also been shown to alter the mammalian circadian clock leading to obesity. In mice, a high-fat diet alters the circadian pattern of feeding and leads to excess calory intake at incorrect circadian time (Bass, 2012). Good health can be maintained by adhering to a fixed feeding schedule that will help preserve and strengthen clock gene expression. Additionally, studies show that evening intake of alcohol, caffeine and nicotine taken as early as 4 h before bedtime is associated with fragmented sleep at night, and therefore should be avoided (Spadola et al., 2019). Short sleep or chronic sleep deprivation leads to disruptions in energy balance and are now considered independent risk factors for metabolic abnormalities like insulin resistance and hyperglycemia (Arble et al., 2015). These studies further highlight the possibility that dietary modifications in circadian rhythms may have therapeutic benefits in the long term.

The ability of the circadian timing system to receive feedback related to alteration of its functioning from exercise or stimulated activity is also well documented. Although light is considered the most important zeitgeber in humans, recent evidence has also highlighted the role of exercise as a chronobiological tool to correct circadian misalignment (Youngstedt et al., 2019). In rodents, stimulated activity leads to a change in SCN properties, neuron firing, and the expression of clock genes (Reebs and Mrosovsky, 1989). The human circadian timing system is responsive to exercise as well. This is evident through the changes in the PRC in humans which describes the relationship between different zeitgeber like light or exercise (the stimulus) and the shift of the circadian rhythms (the response). Recently, the timing of the morning phase advance and the night phase delay regions of the exercise PRC was shown to be comparable to that of bright light PRC (Youngstedt et al., 2019), further emphasizing the pleiotropic role of exercise in correcting circadian misalignment. Exercising causes circadian system timing delay as evidenced by shifts in the timing of melatonin rhythms (Buxton et al., 2003). These responses are linked to exercise intensity and duration which can hasten the circadian rhythms re-entrainment and schedule readjusting while working in shift (Buxton et al., 1997). In geriatric patients, midday long-term fitness training positively consolidates the sleep-wake cycle (Van Someren et al., 1997). Older people with insomnia experience improvement in sleep quality, self-reported, when they exercised during the early evening or afternoon (Reid et al., 2010).

Thermoregulation in our body is another key mechanism that determines the quality of sleep (Gilbert et al., 2004). The principal way the body regulates core body temperature is through a process known as vasodilation and vasoconstriction. Vasodilation increases the blood flow to the extremities which facilitates heat loss when the core temperature is high (Kräuchi et al., 2000). Recent reviews have revealed that heat exposures during sleep can increase wakefulness while drastically reducing REM and slow-wave sleep (Okamoto-Mizuno and Mizuno, 2012). Additionally, humid heat is often associated with an increase in thermal load during the different stages of sleep, which in turn interferes with thermoregulation and affects the quality of sleep (Tsuzuki et al., 2004). While on the other hand, cold exposures don’t seem to affect the sleep stages and therefore don’t affect the quality of sleep even though the cardiac autonomic response may be affected (Okamoto-Mizuno and Mizuno, 2012). Thus, there seems to be an ambient temperature that facilitates good quality sleep. Studies have concluded the ideal bedroom temperature to be approximately 13°C to 23°C when no difference in sleep stages was observed (Muzet et al., 1984). Thus, we can see that the environment where we sleep matters. Noisy environments should also be avoided since it negatively impacts sleep quality (Simons et al., 2018).

Melatonin is a natural hormone that is released by the pineal gland that induces sleep in humans. During the nighttime, there is a surge in the melatonin levels as a result of the inputs received from the SCN. Polymorphisms in melatonin receptors are associated with an increased risk of diabetes, cardiovascular diseases and depression (Lyssenko et al., 2009; Gałecki et al., 2010; Samimi-Fard et al., 2011). Melatonin supplements are readily available and can be used to realign the circadian rhythms with the external environment. Melatonin release from the pineal gland gradually declines with age and is related to lowered sleep efficacy. In older individuals, melatonin supplements have been shown to improve sleep, alertness in the morning and increased cognitive performance (Lemoine and Zisapel, 2012). Melatonin’s role during this pandemic is further highlighted in the repurposing of drugs section described below.

**FIGURE 2:**
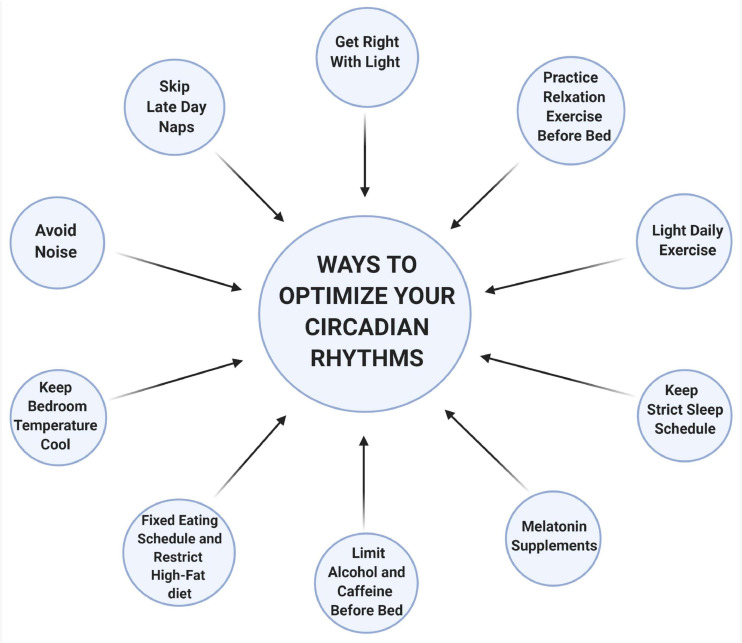
Ways to optimize your circadian rhythms. Light is one of the most important regulators of the circadian rhythms. Chronic exposure to light, particularly to shorter wavelengths (like blue and green), at nighttime inhibits sleep-inducing neurons and decrease melatonin secretion. Naps later during the day usually comprise deep sleep (slow-wave), when there is a natural decline of our energy, and this interferes with nighttime sleep. Hot and humid temperatures increase wakefulness, reduced REM and slow-wave sleep. However, cooler temperatures between 13 to 23°C do not interfere with any of the sleep stages. Noisy environments can also interfere with deep sleep thus a quiet environment is preferred. Food intake at an inappropriate time of the day disrupts the coordinate function of circadian rhythms between the central and the peripheral organs resulting in metabolic imbalances. Alcohol, caffeine, or nicotine should be avoided at least 4 h before bedtime as it is associated with a fragmented sleep pattern. Relaxation exercises before bedtime can help lower heart rate, blood pressure, and reduce sympathetic nervous system activation, which facilitates sleep. Stimulating activities or exercise leads to alteration of the circadian functioning by changing the expression of clock genes and creates shifts in the timing of melatonin rhythms in humans. Melatonin supplements can also help fall asleep, especially during insomnia or jet lag. Adhering to a strict sleep schedule is also important that can help strengthen the circadian rhythms. This schematic is prepared using http://biorender.com.

Circadian rhythms play an essential role in patterning and regulating many physiological functions and gene expression networks within our body. Thus, it is not surprising that the circadian rhythms are sensitive and can be influenced easily by many factors. Studies reveal that 40–70% of the elderly population in the United States experience some form of sleep disturbances (Van Someren, 2000). This is alarming, given the wide implication of a dysregulated circadian rhythms to overall health. Due to the COVID-19 pandemic, it has now become more essential than any time ever that we re-establish alignments to help facilitate proper functioning and robust circadian rhythms. Resetting the circadian rhythms can improve the quality of life, and most importantly help prevent a more serious illness (Figure 2).

## Repurposing Drugs and Chronotherapy for SARS-CoV-2

Despite the advancement in research and collaborative effort worldwide, a viable treatment specific against the SARS-CoV-2 just seems to be out of reach amidst this pandemic. To date, the treatment for patients with COVID-19 mainly incorporates symptoms management only. Researchers across the world are trying to develop new therapeutic drugs to combat the COVID-19. However, designing new drugs is an expensive and time-consuming process. It might take several months to years more to test the efficacy of these drugs, starting *in vitro*, followed by *in vivo* animal models (preclinical testing), and finally testing them in human subjects through controlled clinical trials. Thus, repurposing old drugs for the treatment and management of COVID-19 has become an attractive option. Currently, drugs that are being repurposed fall under one of the two categories: drugs that are able to inhibit/block various stages of the SARS-CoV-2 life cycle in humans, like remdesivir, and those that can counteract the insidious effects caused by the virus. Additionally, some drugs do not fall under any of those two categories but can effectively manage other comorbidities like hyperlipidemia, cardiovascular diseases, pulmonary hypertension, depression, and anxiety in hospitalized COVID-19 patients that are known to increase mortality rates (Li et al., 2020; Richardson et al., 2020; Surveillances, 2020). Some of these classes of drugs may also impart its pleiotropic effect to reduce the severity of symptoms associated with COVID-19.

**FIGURE 3:**
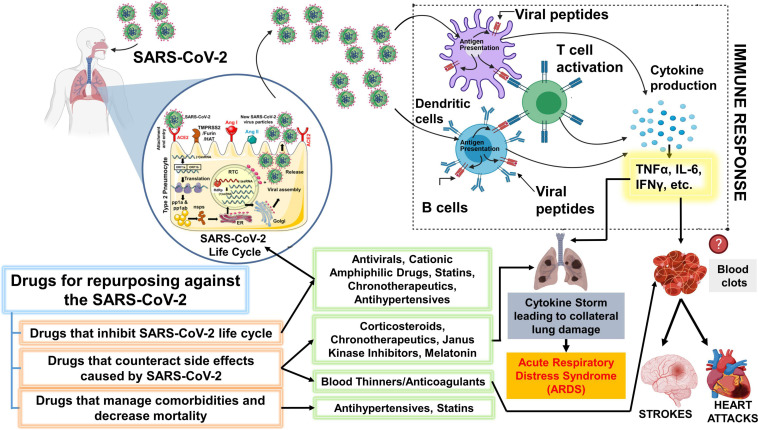
Schematic showing key classes of drugs that are currently repurposed and suggested drugs that could be repurposed for SARS-CoV-2. The drugs proposed for repurposing have been classified under 3 categories: Drugs that inhibit SARS-CoV-2 lifecycle, counteract the side effects post-infection or can efficiently manage comorbidities and other risk factors. It is important to note that the representation above is purely hypothetical, and based solely on the limited literature that was available on SARS-CoV-2 or the beneficial effects that were observed with other viruses from both *in vitro* or *in vivo* studies. Antiviral drugs like remdesivir are effective against the SARS-CoV-2. Statins have also been shown to inhibit the SARS-CoV-2 life cycle. Cationic amphiphilic drugs (CADs), like amiodarone, chlorpromazine, and clomiphene have been shown to inhibit several virus families including the SARS-CoV-1 and MERS-CoV. Therefore, CAD’s potential against the SARS-CoV-2 should be further evaluated. Janus-associated kinase inhibitors, baricitinib, have been shown to decrease mortality among COVID-19 patients when used together with remdesivir. However, the beneficial effect of common corticosteroids like prednisone or methylprednisolone has not been determined but several clinical trials are evaluating the same. A higher incidence of blood clots is observed among COVID-19 patients. The massive inflammatory response during SARS-CoV-2 infection can trigger blood clots which can eventually cause strokes or heart attacks. Thus, blood thinners, which have been associated with decreased mortality among COVID-19 patients can be beneficial. We have additionally proposed a novel chronotherapeutic agent, SR9009, which has immense potential to inhibit several virus families, reduce lung inflammation and injury by interfering with the production of inflammatory cytokines. Additionally, REV-ERB agonists (e.g., SR9009 and GSK2667) can also be shown to boost endurance, reduce cholesterol, body weight and prevent heart disease. This schematic was prepared using SMART (Servier Medical Art), licensed under a Creative Common Attribution 3.0 Generic License. http://smart.servier.com/ and http://biorender.com.

Chronotherapy is an emerging focus area that utilizes the approach of delivering drugs at specific times of the day to either maximize treatment efficacy or minimize negative effects (Zaki et al., 2019). Recent report has demonstrated that circadian clock targets Bmal1 and Rev-erbα both influence the life cycles and replication of Hepatitis C virus and other related Flavivirus (e.g., Dengue and Zika virus) by modulating viral receptors in a human hepatoma cell line (Huh-7) (Zhuang et al., 2019). Additionally, researchers showed that genetic ablation of Bmal1 and overexpression or over activation of Rev-erbα using synthetic ligands such as GSK2667 and SR9009 blocks HCV viral replication through perturbation of lipid signaling pathways (Zhuang et al., 2019). Understanding the novel role of circadian clock components such as Bmal1 and Rev-erbα, and repurposing novel chronotherapeutic drugs (e.g., Rev-erbα agonists) for the treatment of SARS-CoV-2 and other respiratory viral infections will help reduce the exacerbation events that occur in patients with chronic lung diseases and thereby reduce the number of deaths caused by epidemic/pandemic respiratory viral infections worldwide (Figure 3). Previously, a circadian gene expression atlas in mammals revealed the importance of circadian biology in medicine. This study showed that 56 of the top 100 best-selling drugs, directly or indirectly, target a product of a circadian gene (Zhang et al., 2014). Approximately half of these drugs show a very short half-life (<6 h), suggesting the importance of timed-administration and its impact on mode of action (Zhang et al., 2014). Here, we will examine the chronotherapeutic advantages of some major classes of drugs that can be repurposed for the treatment and management of COVID-19 and highlight some of the knowledge gaps in this domain for the scope of future research.

## Hyperlipidemia Drugs

Statins are used widely for the prevention of cardiovascular events by reducing *in vivo* cholesterol synthesis (Ziaeian and Fonarow, 2017). These classes of drugs can potentially ward off uncontrolled systemic inflammatory response triggered by SARS-CoV-2 in COVID-19 patients. The anti-inflammatory effects of statins can block the infectious potential of enveloped viruses, as *in vitro* studies have shown, and therefore statins are now used by clinicians as a part of their treatment protocol (Schönbeck and Libby, 2004; Massachusetts General Hospital, 2020). If lipophilic statins reach the site where the virus accumulates and unleashes damage, it can considerably lessen the mortality risk among COVID-19 patients (Rossi et al., 2020). Additionally, statins have been independently associated with lower ICU admission among hospitalized COVID-19 patients (Tan et al., 2020; Figure 3). Statins reduce hyperlipidemia and bring about a reduction in cytokines with its pleiotropic effects under different non-infective conditions (Wassmann et al., 2003; Fang et al., 2005). In a randomized controlled trial, atorvastatin was evaluated for treating influenza virus infection and the results showed remarkable potential in lowering inflammatory cytokines level [NCT02056340], which often causes damage to vital organs during hyperinflammation.

Cholesterol biosynthesis follows a strict circadian pattern, which typically peaks at night between 12:00 and 6:00 am, during which the 3-hydroxy-3-methyl-glutaryl-coenzyme A reductase (HMG-CoA reductase) the rate-limiting enzyme of cholesterol biosynthesis peaks (Izquierdo-Palomares et al., 2016). When hypolipemic therapy is adjusted and aligned with biological rhythms, the efficacy has been determined to be better in several studies. Particularly, the administration of statins in the evening is more effective than when it is taken in the morning (Saito et al., 1991; Lund et al., 2002; Wallace et al., 2003; Ozaydin et al., 2006; Tharavanij et al., 2010). This makes sense when we realize that cholesterol synthesis peaks during midnight. Additionally, when using statins, the half-life of the drug should also be carefully considered. For instance, simvastatin has a very short half-life of 2–3 h, compared to Atorvastatin or rosuvastatin, which has a half-life of 14 and 30 h, respectively. Therefore, the time of administration becomes even more important for simvastatin compared to others. However, studies have also reported better outcomes in atorvastatin with evening administration (Ozaydin et al., 2006). Thus, when one dose of statins is administered daily in the evening, its inhibitory activity seems to be expressed maximally since cholesterol biosynthesis happens optimally at that nighttime. Clinicians should therefore take the timing of statin administration into account, which can subsequently lower drug toxicity in COVID-19 patients. Future research will focus to determine the efficacy of timed-administration of statins in COVID-19 patients.

## Blood Thinners/Anticoagulants

In the early phase of the pandemic, clinicians observed an increased rate of blood clots among the hospitalized COVID-19 patients (Malas et al., 2020). This helped explain the increased incidence of critical complications such as lung failure, stroke, and myocardial infractions among the COVID-19 patients (Morrone and Morrone, 2018). Although the exact cause of this increased incidence of blood clots among COVID-19 patients remains unclear, evidence suggests that severe inflammatory response, similar to that of COVID-19 patients with a hyperactive immune system, can trigger coagulation, decrease the activity of natural anticoagulants and also impair the fibrinolytic system (Esmon, 2005). However, whether it was safe to administer anticoagulants without any consequences were not determined earlier. In a recent large clinical study, comprising of patients from more than 300 hospitals worldwide, it was established that full dose anticoagulants (blood thinner) when given to moderately ill hospitalized COVID-19 patients, the requirement for vital organ support such as ventilation was significantly reduced. In addition to being safe, a full dose of blood thinners gave superior results in preventing ICU admits when compared to the dose normally given to patients to prevent blood clots (National Institutes of Health, 2021). Additionally, prophylactic anticoagulation administration did not increase the risk of serious bleeding in COVID-19 patients (Rentsch et al., 2021). Translational researchers are still trying to further optimize the administration of these drugs to improve the quality of care provided to patients suffering from COVID-19 (Figure 3).

One such optimization technique can consider the timing of the drug administration and the half-life of these drugs. Diurnal variation in coagulation and fibrinolysis have been observed in humans in the past. Hypercoagulatory and hypofibrinolytic conditions are more frequent in the morning than any other time during the day because of increased platelet activity. Between 8 to 10 am, the concentration of coagulation factors such as Factor V, VII, prothrombin fragment F_1 + 2_, and D-dimer peaks (Kapiotis et al., 1997). Additionally, the activity of the plasminogen activator, which facilitates fibrinolysis, is highest only in the afternoon (Angleton et al., 1989). This partly explains the increased incidence of thromboembolic events during the early hours of the day. Rivaroxaban (Xarelto in the United States) is a commonly prescribed blood thinner that is an inhibitor of activated coagulation factor X and has a very short half-life of 5–9 h and does not accumulate even with multiple doses (Kubitza et al., 2005; Mueck et al., 2014). It is administered once a day and used for multiple purposes like lowering the risk of strokes, deep vein thrombosis, pulmonary embolism, and other conditions. A study observed that rivaroxaban concentration was significantly higher 12 h after evening intake than it was when taken in the morning (53.3 ng/mL vs. 23.3 mg/mL). Additionally, the evening regimen was able to significantly reduce the prothrombin fragment F_1 + 2_ concentration better in the morning when compared to the morning regimen (85 ± 25 nmol/L vs. 106 ± 34 nmol/L), and also evening intake had a longer-lasting effect (Brunner-Ziegler et al., 2016). Similar effects have been observed with another antiplatelet drug, Aspirin. Van Dieman et al. examined the effect of morning vs. evening administration of aspirin on the platelet activity in patients with stable cardiovascular disease and found that evening regimen resulted in higher platelet inhibition and a significant reduction in reticulated platelets compared to the morning regimen (van Diemen et al., 2020). Another study showed that bedtime administration of aspirin significantly reduced platelet reactivity (compared to morning administration) during the morning high-risk hours but did not change the blood pressure in patients using aspirin for cardiovascular diseases (Bonten et al., 2015).

While a plethora of literature is not available for all types of blood thinner, there is sufficient evidence that proves circadian variation in coagulation and fibrinolysis exists, and certain anticoagulatory or antiplatelet drugs work best only when administered at a specific time of the day. Therefore, timed-administration of these drugs can tremendously prove to be a cost-effective way to improve patient care during these times with the added benefit of reducing drug toxicity while maximizing the benefit in critically ill COVID-19 patients. Future studies should therefore aim to further evaluate the effect of timed-administration of full doses of blood thinners, once a day, on COVID-19 patients.

## Antihypertensive Drugs

Earlier, links were seen between hypertension and COVID-19 fatalities (Huang S. et al., 2020). This was ascribed to the relationship between SARS-CoV-2 and angiotensin-converting enzyme (ACE2). ACE2 was purportedly serving as an entry port for the SARS-CoV-2 (Davidson et al., 2020). Yet some evidence indicated that COVID-19 induced acute lung damage can be reduced by renin-angiotensin-aldosterone system inhibitors and can be beneficial in hypertensive hospitalized COVID-19 patients (Baral et al., 2020). At the European Society of Cardiology Congress 2020, the BRACE CORONA trial’s data showed zero difference in results among patients who continued consuming ARBs (angiotensin receptor blockers) or ACE inhibitors and those who didn’t consume them for a month after being diagnosed with COVID-19 (European Society of Cardiology, 2020). The first randomized trial suggests that heart patients who take ACE and ARBs can safely consume them even after contracting COVID-19 (Figure 3).

Blood pressure (BP) control laxity in United States adults is increasing hypertension, an important modifiable risk factor for strokes and heart attacks (Fuchs and Whelton, 2020). Patients with severe heart problems and hypertension have enhanced risk if they contract SARS-CoV-2. A large study from China revealed that overall case fatality from COVID-19 was 2.3%, but it increased to 6.0% for hypertensive patients (Surveillances, 2020). Studies have documented diurnal changes in blood pressure are higher during the early morning and lower in the late evening or sleeping hours. This pattern is more prominent in dippers, those who experience a 10 to 20% reduction in their nocturnal BP, and a slightly lesser extent on non-dippers, those that experience a blunted decline in their BP at night (Di Raimondo et al., 2020). Increased night-time ambulatory blood pressure, as studies suggest, is related to fatal and non-fatal cardiovascular events like myocardial infarction, cardiac arrest, etc. (Elliott, 1999). Therefore, lessening BP at night is important from the perspective of bringing down cardiovascular events particularly in hypertensive, hospitalized COVID-19 patients. Improvement in 24 h BP was observed along with a dip in BP profile when a minimum of one antihypertensive drug was administered in the evening (Bowles et al., 2018). Hermida et al. studied the chronotherapeutic effect of morning vs. evening administration of antihypertensive drugs and found that bedtime administration, as opposed to morning, significantly reduced BP throughout the night and in the morning hours, and markedly reduced the likelihood of a major cardiovascular event (Hermida et al., 2020). Other antihypertensive drugs, like ramipril, telmisartan, amlodipine with hydrochlorothiazide, amlodipine with olmesartan, and valsartan have also been shown to have better effects when taken in the evening or before bedtime (Hermida et al., 2007; Hermida and Ayala, 2009; Hermida et al., 2010; Hoshino et al., 2010; Zeng et al., 2011). However, antihypertensive drugs like amlodipine, which has a long half-life of 35–50 h, made no difference between evening or morning administration (Mengden et al., 1993; Nold et al., 1998). Overall, the results from several human studies have shown that ARBs and ACE inhibitors are completely safe for consumption in hypertensive patients. Additionally, hypertensive medications have also been associated with decreased mortality among COVID-19 patients. Thus, these drugs can be potentially effective remedies for the treatment and management of hypertensive COVID-19 patients. We additionally advocate taking into consideration the time of administration of these hypertensive drugs for maximum benefit.

## Antiviral Drugs

In October 2020, 7 months since the declaration of the COVID-19 pandemic, the United States FDA authorized the antiviral drug, remdesivir to treat COVID-19. Earlier, remdesivir was shown to be effective against viruses in other coronavirus families like the Middle East Respiratory Syndrome coronavirus (MERS-CoV) and severe acute respiratory syndrome coronavirus (SARS-CoV) (de Wit et al., 2020; Malin et al., 2020). Additionally, studies have also observed remdesivir to have a superior effect over placebo in shortening the recovery time in hospitalized COVID-19 patients (Madsen, 2020). This facilitated the use of remdesivir by clinicians among hospitalized COVID-19 patients. However, a recent WHO solidarity trial published, taking 11,330 adult COVID-19 patients from 30 countries worldwide, showed that antiviral drugs like remdesivir and lopinavir have little to no effect on the outcome such as overall mortality, the need for ventilation support, or hospital recovery time (WHO Solidarity Trial Consortium, 2020; Figure 3 and Supplementary Table 1). Yet, remaining optimistic amidst this deadly pandemic and tweaking strategies in antiviral drug administration may help with better outcomes in vulnerable patients.

Viruses are obligate parasites that rely totally on hosts to survive, replicate and disseminate. The viral life cycle starts with the viral entry into the cell through binding to host receptors or factors manifest at the cell surface. Once the virus enters the cell and capsid disassembly happens, the release of DNA or RNA genomes occur. The translational and transcriptional pathways in a host are exploited for initiating viral replication (Ryu, 2017). About 80% of protein-coding genes in different tissues, as shown in a study on primates, exhibit rhythmic expression on a daily basis (Mure et al., 2018). Host clock components help viruses replicate in a direct or indirect manner. A study found that when wild-type mice, living in a controlled environment with 12 h of light-dark cycles, were infected with herpes virus at the start of the day (at sunrise, when nocturnal animals are transitioning into their resting phase), the viral load was 10 times greater than those infected 10 h after sunrise (around the time when nocturnal animals are starting their active phase). On the other hand, when the researcher knocked out Bmal1, a core circadian clock gene, the level of viral replication remained the same (Edgar et al., 2016). This study further reiterated that circadian variation in immune response or viral activity exists. However, timed-administration of antivirals in patients has rarely been taken into consideration. In a recent retrospective study, the effect of administering antiviral drugs in the morning and evening was evaluated on COVID-19 patients in Italy. The result indicated that CRP values reduced significantly for the morning regimen although measurable differences were not evident in other evaluated parameters (De Giorgi et al., 2020). In another study, mice dosed with acyclovir to prevent HSV-2 infection during the active phase were four times more compared to the resting phase (Matsuzawa et al., 2018). The half-life of a drug is linked to dosing time. Hence, drugs having short half-life of 6 h or less exhibit higher sensitivity to the time during the day when they are administered (Sahin and Benet, 2008). Timed-administration of drugs is a promising approach that needs to be investigated to optimize the available antiviral drugs, like remdesivir and other antiviral drugs that can be repurposed for the treatment and management of COVID-19 patients (Supplementary Table 1).

## Corticosteroids

Recommendations regarding the use of corticosteroids for the treatment and management of COVID-19 largely stemmed from a clinical trial conducted in the United Kingdom, where dexamethasone, a corticosteroid, was observed to reduce mortality at 28 days in hospitalized COVID-19 patients (RECOVERY Collaborative Group, 2020a). Corticosteroids are anti-inflammatory drugs that work by suppressing the activation of the immune system, thus preventing the so-called “cytokine storm” in patients with a hyperactive immune response (Channappanavar and Perlman, 2017). Cytokine storms are responsible for the damage to vital organs like the lungs, which is mainly responsible for COVID-19- related deaths (Ruan et al., 2020). Dexamethasone, prednisone, and methylprednisolone are potent anti-inflammatory drugs that could help prevent these deleterious effects. A recent meta-analysis of the clinical trials involving the use of corticosteroids revealed that compared to the control (placebo), treatment with corticosteroids significantly reduced the risk for mortality (Sterne et al., 2020; Figure 3 and Supplementary Table 1).

Nevertheless, the risks and benefits of using corticosteroids in COVID-19 patients have to be further evaluated carefully before its implementation on a larger scale. Particularly, the use of prednisone or methylprednisolone cannot be blindly implemented since dexamethasone has shown some positive outcomes and have similar anti-inflammatory effects. It is important to note that routine administration of corticosteroids can exacerbate COVID-19-induced lung injury in some instances (Russell et al., 2020). Corticosteroids are only beneficial in cases of hyperinflammation. Earlier, prednisone has been shown to have both beneficial and detrimental outcomes in patients with critical pulmonary infections. For instance, while patients suffering from Pneumocystis jirovecii Pneumonia benefited from using prednisone (with a reduced death rate), patients with MERS-CoV infection experienced a delay in their viral clearance (Bozzette et al., 1990; Arabi et al., 2018). Similarly, in influenza virus-induced severe cases of pneumonia, the use of corticosteroids has also been reported to have worse outcomes (Rodrigo et al., 2016).

Indeed, treatment with corticosteroids in patients suffering from ARDS has shown some conflicting results. Yet, a meta-analysis of several clinical trials has shown that overall corticosteroids reduce the risk of mortality and the duration of mechanical ventilation in patients suffering from ARDS (Mammen et al., 2020). There are still controversies regarding the use of corticosteroids for COVID-19 treatment, particularly because of the absence of the larger randomized clinical trials. Corticosteroids like dexamethasone, prednisone, or methylprednisolone are used in clinical medicine because they possess immunosuppressive effects. Thus, these drugs may have adverse effects in COVID-19 patients if used in full doses (Russell et al., 2020). Therefore, timed-administration of the drugs should be carefully considered in this realm. In patients with rheumatoid arthritis (RA), the chronotherapeutic use of prednisone has been prominently highlighted. Diurnal variation in pain, stiffness and functional disability has been observed in patients suffering from RA, with a peak around the morning hours as a result of an elevated level of pro-inflammatory cytokines throughout the nighttime (De Silva et al., 1984). Prevention of this nighttime rise in proinflammatory cytokines, particularly, IL-6, by evening doses of prednisone, is hypothesized to be better for RA patients than managing morning symptoms when it becomes too late to curb the inflammation. Delayed-release prednisone tablets, which are modified to be released slowly in the blood and many hours after the intake, have been developed so that patients do not have to get up at night to take their medications. These modified drugs were significantly more beneficial to RA patients (Buttgereit et al., 2008). Studies have repeatedly shown that evening administration of prednisone was more beneficial in reducing RA symptoms in patients when compared to the same dose taken in the morning (De Silva et al., 1984). Thus, it remains vital to consider timed-administration of corticosteroids as a potential variable when evaluating the efficacy of corticosteroids against COVID-19 (Supplementary Table 1). To date, there are no published clinical studies that have evaluated the efficacy of prednisone or methylprednisolone against the SARS-CoV-2, and therefore future studies should aim to address this knowledge gap.

## Chronotherapeutic Drug/REV-ERBα Agonists

Almost all living organisms from bacteria to humans possess an internal clock that oscillates rhythmically to help anticipate environmental changes (Patke et al., 2020). In mammals, this is possible through a translational-transcriptional feedback loop that regulates a gene expression pattern. The dimerization of two transcription factors, CLOCK and BMAL1, followed by the binding of the CLOCK-BMAL1 complex at the E-box motif in the promoter region of the core clock genes, facilitate the transcription of Period genes (Per1, Per2, and Per3) and Cryptochrome genes (Cry1 and Cry2). In turn, PER-CRY protein complex inhibits the transcription of its own genes by directly inhibiting CLOCK:BMAL1 activity. In addition to this, there are two nuclear receptors involved in this feedback loop known as the REV-ERBα and RORα. RORα work against REV-ERBα to facilitate the transcription of Bmal1 by binding to the promoter region (Sundar et al., 2015b,c). Thus, both REV-ERBα and RORα plays a crucial role to maintain the circadian clock machinery.

SR9009, a REV-ERBα agonist, is a synthetic drug that was originally designed to better understand circadian rhythms *in vitro* and *in vivo*. In mice, this drug was shown to boost endurance by increasing mitochondria count in skeletal muscles (Woldt et al., 2013), significantly reduced cholesterol and body weight (Solt et al., 2012), and also lower anxiety to a level as effective as using benzodiazepine (Banerjee et al., 2014). SR9009 was effective in reducing inflammation by interfering with the production of inflammatory cytokines like TNFα, CCL2, and MMP-9 in a rat model (*in vivo*) and rat C6 astroglial cells (*in vitro*) (Li et al., 2014; Morioka et al., 2016). Additionally, some studies have also observed some great benefits of using SR9009 treatment for heart disease. For instance, when genetically modified mice prone to blood vessel lesions and hardening of arteries were administered with SR9009, there was a significant reduction in observed lesions while other parameters mostly remained unchanged (Sitaula et al., 2015).

Circadian rhythms have been shown to regulate both the innate and adaptive immune systems in multiple ways. Although the exact mechanisms are not known, it has been determined that changes in core clock genes and the nuclear factors can increase the host’s susceptibility to infectious agents (Scheiermann et al., 2013). For instance, the genetic ablation of the Bmal1 and the stimulated activation of REV-ERBα with synthetic agonist (like SR9009) was successful in inhibiting the positive-strand RNA viruses of the Flaviviridae family like the Hepatitis C, Dengue, and the Zika virus by interfering with the lipid signaling pathway (Zhuang et al., 2019). Additionally, REV-ERBα agonists were also shown to regulate HIV-1 replication by inhibiting promoter activity in CD4 T cells and macrophages, while antagonism of REV-ERBα lead to increase promoter activity (Borrmann et al., 2020). Yet another evidence showed that SR9009 was successfully able to inhibit replication of alphaviruses like chikungunya and o’nyong’nyong virus by suppressing the synthesis of the structural proteins (Hwang et al., 2018). Besides the drug’s ability to inhibit the aforementioned virus, COVID-19 patients might benefit the most from its potential to selectively regulate pro-inflammatory cytokines like IL-6, which has been considered as a prognostic marker for mortality among the same (Gibbs et al., 2012; Liu et al., 2020). Considering the beneficial effects of REV-ERBα agonists, like SR9009, which was originally developed to study circadian rhythms, can therefore be implemented as a unique therapeutic drug against the SARS-CoV-2 during these unprecedented times. However, the chronotherapeutic drugs such as REV-ERBα agonists should be initially tested using *in vitro* and *in vivo* models of SARS-CoV-2 infection before they can be repurposed to the treatment of COVID-19 (Figure 3 and Supplementary Table 1).

## Cationic Amphiphilic Drugs

Cationic amphiphilic drugs (CADs) belong to a wide group of compounds that share several structural properties like a hydrophobic ring or ring system on the molecule and possess a hydrophilic side chain with an ionizable cationic amine group. CADs may include drugs used for the treatment of depression, psychosis, bacterial infections, arrhythmias, malaria, and lower cholesterol (Halliwell, 1997). Some of these drugs are effective against several infectious agents including viruses. For instance, the antimalarial drug Hydroxychloroquine and Chloroquine were found to be successful in preventing the SARS-COV-2 virus replication *in vitro*. For this reason, clinicians were hopeful that the *in vitro* results would translate *in vivo* and started using those antimalarials for the treatment of COVID-19 worldwide (Giri et al., 2020). However, larger clinical trials had clearly shown that these drugs were not effective against the SARS-COV-2 in humans (RECOVERY Collaborative Group, 2020b). Fortunately, other FDA-approved CADs might be useful (Figure 3 and Supplementary Table 1).

Anti-arrhythmic drug, amiodarone is an ion channel blocker used for the treatment of ventricular arrhythmias and atrial fibrillation. This drug can prevent the fusion of the viral envelop with the endosomal membrane and also accumulate in the late endosomes/lysosomes and disrupts the viral endocytic pathway (Salata et al., 2015). Earlier amiodarone was shown to prevent the entry of the Filovirus at concentrations that are found in the patient’s serum routinely using anti-arrhythmic drugs (Gehring et al., 2014). A similar result was also observed with the Ebola virus using methyldiethanolamine, a metabolite product of amiodarone (Salata et al., 2015). Amiodarone, dosed at 60 mg/kg, significantly increased survival in mice infected with the ebola virus (Madrid et al., 2015). However, amiodarone wasn’t effective against the Ebola virus in humans in the Western African population (Wolf et al., 2015). Amiodarone was also shown to inhibit other viruses like the arenavirus, the SARS-CoV-1 and Hepatitis C virus (Stadler et al., 2008; Cheng et al., 2013; Gehring et al., 2014). Arrhythmias are common among COVID-19 patients, and it is associated with higher morbidity and mortality (Babapoor-Farrokhran et al., 2020). Amiodarone might be particularly beneficial for COVID-19 patients showing signs of ventricular arrhythmias, although the pros and cons of these drugs need to be studied in detail before using for the treatment of COVID-19 patients. The time of the day when the occurrence of cardiac arrhythmias is more frequent has not been studied extensively and multiple studies investigating the same have failed to take potential confounders like alcohol or caffeine consumption into consideration. Thus, interpreting circadian patterns in arrhythmias have been difficult. However, Portaluppi and Hermida summarized the available literature, and most studies found a peak time occurrence of cardiac arrhythmias (Ventricular pre-mature beats, ventricular tachycardia, or fibrillation) mostly between 6 am and noon. Based on their findings, they concluded that arrhythmogenesis appeared to be less frequent or suppressed during the nighttime (Portaluppi and Hermida, 2007). This information can be useful for clinicians who are treating COVID-19 patients with cardiac arrhythmias. Future research is needed in this domain to evaluate the consequence of timed-administration of anti-arrhythmic drugs.

Another class of CADs is the antipsychotics, particularly chlorpromazine, which has been shown to have antiviral properties against the Crimean-Congo hemorrhagic fever virus, the adenovirus, Ebola, MERS-CoV and SARS-CoV (Bhattacharyya et al., 2010; Diaconu et al., 2010; Dyall et al., 2014; Ferraris et al., 2015). During the first stage, the endocytosis of the virus, clathrin-coated pits are internalized along with the virus and forms the clathrin-coated vesicles (Bayati et al., 2021). Chlorpromazine blocks the formation of clathrin-coated pits and thus prevents viral entry into the cells (Daniel et al., 2015). Several other antipsychotic drugs have also been reported to have antiviral properties. For instance, antidepressant drug desipramine has been shown to prevent parvovirus entry into cells by introducing disorder in the cholesterol-rich lipid rafts (Pakkanen et al., 2009), and sertraline has been reported to inhibit the Zika virus (Barrows et al., 2016). Nagayama et al., studied the effect of timed-administration of chlorpromazine in rats and found that the sedation period of the drug was both time- and dose-dependent. In rats, housed in a controlled environment with a 12-h light-dark cycle (19:30 – lights on, 7:30 – lights off), at 1:30 a significantly smaller dose of chlorpromazine was able to show the same sedative effect than when administered at 7:30, and the response increased with increasing dose. The sedation period variation was attributed to the sensitivity of the catecholamine receptors but the exact reasons are not known (Nagayama et al., 1978;Supplementary Table 1). The purpose of using chlorpromazine for COVID-19 is a different purpose altogether. However, this study might suggest that there may be a specific window of the day when the drug might be best utilized. Ongoing and future studies can attempt to see if there is any variation in time-dependent response.

Another class of drugs belonging to the CADs is the selective estrogen receptor modulators (SERMs). SERMs can exert both agonist and antagonist effects depending on the location of the estrogen receptor. In the breast, estrogen receptors are different from that of the other parts of the body such as the bone, liver, or uterine cells. SERMs block estrogen function in the breast while activates it on the other parts of the body (Riggs and Hartmann, 2003). SERMs have earlier been shown to have protective functions against MERS-CoV, Ebola virus, HSV-1, and HCV (Johansen et al., 2013; Murakami et al., 2013; Zheng et al., 2014). SERM, clomiphene, possesses antiviral activity against the Ebola virus by interfering with the late-stage fusion of the viral envelope with the endosomal membrane (Nelson et al., 2016). Similar to the Ebola virus, the SARS-CoV-2 is a lipid-enveloped virus that encounters the host late endosome/lysosome during its replication cycle. Additionally, clomiphene has also been shown to inhibit the Ebola virus entry by inhibiting the NPC1-dependent pathway, which has been hypothesized to increase cholesterol accumulation in the late endosomes and impair viral entry for SARS-CoV-2 (Ghasemnejad-Berenji et al., 2020). Overall, CADs are effective against many viruses *in vitro*. CAD’s ability to interfere at different stages of the viral life cycle may be exploited to see if any benefit exists against the novel SARS-CoV-2.

## Janus-Associated Kinase Inhibitor

In the severe cases of COVID-19, the overactivation of the immune system, followed by a cytokine storm results in profound damage to vital organs, especially the lungs. This causes the development of ARDS, the leading cause of death among COVID-19 patients (Huang C. et al., 2020; Zhou et al., 2020). Proinflammatory cytokines, particularly IL-6 through the activation of the Jak-Stat pathway, have been identified as a prognostic indicator for mortality among COVID-19 patients (Del Valle et al., 2020; Liu et al., 2020). Therefore, known drugs capable of inhibiting the Jak-Stat pathway can be beneficial in patients suffering from COVID-19. JAK inhibitors can prevent the phosphorylation of proteins that are involved in the signal transduction cascade of the Jak-Stat pathway and thus are able to reduce cytokine-mediated inflammation and collateral damage to vital organs (Lin et al., 2020).

Baricitinib is a JAK inhibitor used for the treatment of rheumatoid arthritis (Lin et al., 2020). Recently, this drug has received some attention after a study showed that baricitinib significantly reduced the median number of days to recovery, from 18 to 10 days, in hospitalized COVID-19 patients requiring high-flow oxygen or non-invasive ventilation, when used together with remdesivir (Kalil et al., 2020). Baricitinib was very well tolerated among COVID-19 patients and had a significant reduction in inflammation as measured by the levels of inflammatory cytokines. Another study showed that the need for ventilation support or death was also reduced by more than 50% (34.9 to 16.9%) using baricitinib compared to the control group (Stebbing et al., 2021). At clinically relevant concentration, Baricitinib can successfully inhibit type-1 interferon response (exaggerated in COVID-19 patients) which is known to increases ACE2 expression (in liver cells), the same cell receptor used by the SARS-CoV-2 to enter host cells, increasing the viral load (Stebbing et al., 2021). Additionally, baricitinib is a potent inhibitor of the Numb-associated kinase (NAK) family of proteins, particularly AAK1, that plays a pivotal role in clathrin-mediated endocytosis, which further prevents viral entry into the cells (Conner and Schmid, 2002; Sorrell et al., 2016). Despite promising findings in smaller studies, sufficient evidence is still lacking that demonstrates the beneficial effects of baricitinib particularly against the SARS-CoV-2. We, therefore, advocate for larger clinical trials and *in vivo* animal studies that will help to determine the optimal dose and efficacy of these drugs. Other JAK-inhibitors like fedratinib and ruxolitinib can also be explored at the same time for their efficacy against the SARS-CoV-2 (Figure 3 and Supplementary Table 1).

Timed-administration should also be taken into consideration when using Baricitinib. This is because this drug is metabolized rapidly in the body and has a very short half-life in humans (Kim et al., 2018). In collagen-induced arthritis mice models, the diurnal variation in the expression of inflammatory cytokines was observed. Subsequently, the superior effect of baricitinib was observed when the drug was administered in a period (evening) where the cytokine production was the highest (Yaekura et al., 2020). Similarly, diurnal variation of the pro-inflammatory cytokine, IL-6, has been observed in humans (Gudewill et al., 1992). In healthy volunteers, IL-6 levels correspond with the sleep-wake cycle (Bauer et al., 1994). Many studies have observed a peak of IL-6 levels during nighttime and early morning hours (Vgontzas et al., 2005). However, how these pro-inflammatory cytokine levels change during SARS-CoV-2 infection is not well known. Future studies should aim to determine the diurnal changes of proinflammatory cytokines, if possible, in COVID-19 patients, at different time points during the day. These studies can further determine if the timed-administration and slow release of these anti-inflammatory drugs, like Baricitinib, can lead to even better outcomes in COVID-19 patients.

## Melatonin and Circadian Rhythms

Melatonin is the hormone produced in the pineal gland during the dark (night), and its synthesis is directly controlled by the central clock (SCN) (Blask, 2009). Melatonin exerts its effects by binding to one of two melatonergic receptors, namely MT1 and MT2, both of which belong to the G protein-coupled receptor family. Both receptors have the same affinity for melatonin, but the MT2 receptor has double the dissociation half-time of MT1 (Legros et al., 2014). Expression of MT1 in retinas, kidneys and the SCN implicates the role of both melatonin and MT1 in the regulation of circadian rhythms and reproductive cycles. Expression of the MT2 subtype is somewhat similar to that of MT1 in areas such as the retina and brain, but MT2 is completely absent in the SCN. The MT2 melatonin receptor plays a major role in the regulation of body temperature (Pandi-Perumal et al., 2008; Bjorvatn and Pallesen, 2009). Circadian regulation of melatonin signaling is one of the ways by which it influences sleep timing. Studies that utilize exogenous melatonin for the treatment of age-related sleep disorders demonstrate how melatonin can have profound effects on sleep and circadian rhythms.

Prior report has shown that administration of a physiologically relevant dose of melatonin helps improve sleep quality in older individuals who generally demonstrate poor sleep quality (Luboshizsky and Lavie, 1998; Zhdanova et al., 2001). Using an *ex-vivo* approach, melatonin treatment in brain SCN slices from WT mice resulted in inhibition of neuronal firing in a concentration-dependent manner. Alternatively, this process was inhibited in brain SCN slices from MT1 knockout mice treated with melatonin, indicating that MT1 has a profound effect on the central clock (Dubocovich, 2007). Thus, melatonin may serve as one of the key modulators of sleep, circadian rhythms, and immunity in the elderly. The previous report showed rhythmic changes in circulating immune cells and leukocyte migration from the blood to various organs. Bmal1 deletion in endothelial cells resulted in an abolished time of day-dependent differences in expression of VCAM-1 and ICAM-1, and failure to maintain rhythmic homing of immune cells into peripheral tissues (He et al., 2018). In another report, the role of melatonin controlling circadian clock target Bmal1 occurs via activation of PI3K/AKT signaling that is critical for cell survival (Beker et al., 2019). Melatonin (10 mg/kg/d, s.c for 7 days) treated aged male Wistar rats (28 months old) when given 4 × 10^8^ sheep erythrocytes via i.p. evoked augmented humoral immune response (IgG1 and IgM levels) compared to aged controls. This finding suggests that exogenous melatonin treatment showed attenuation of humoral immune responses in aged rats (Akbulut et al., 2001). Therefore, it is vital to unwrap the novel relationship between endogenous or exogenous melatonin signaling and circadian rhythms that may be directly contributing to outcomes of SARS-CoV-2 infection. Future and ongoing studies will address the potential role of the host circadian clock-aging-immunity axis on the pathogenesis of SARS-CoV-2-induced viral infection.

Studies also indicate that melatonin levels gradually decrease with age, causing disruptions of many circadian controlled physiological functions (Karasek, 2004). Therefore, melatonin supplements can be useful that have the potential to reverse a dysfunctional circadian rhythms. Additionally, melatonin is a potent antioxidant and possesses immunomodulatory, antiviral, and anti-inflammatory properties. A detailed explanation of the different properties are beyond the scope of this review but can be found here (Malhotra et al., 2004; Anderson and Reiter, 2020). In the elderly population, the lower level of melatonin therefore partially explains why certain age groups are more likely to suffer from more severe symptoms of the COVID-19 than the younger generation. The use of melatonin supplements has been shown to reduce severity in other viruses and may be useful for COVID-19 patients as well (Figure 3). To date, there is no randomized control clinical trial conducted using melatonin for the treatment of COVID-19 patients is available. Thus, we advocate for future clinical studies that will aim to address the benefits of using melatonin supplements in elderly COVID-19 patients.

## Vaccinations

As of February 20, 2021, approximately 12.6 percent of the United States population has received at least one dose of either the Moderna or the Pfizer mRNA vaccine, and about 4.9 percent are fully vaccinated. Currently, the United States is administering about 1.9 million doses per day (Johns Hopkins University and Medicine, 2021). While it is true that mass vaccination could be the only answer to eradicate the SARS-CoV-2 from the globe, as we have seen in the past with smallpox and the poliovirus in the United States, it might take longer time than we imagine, especially with the slow progress and the newly emerging variant of SARS-CoV-2 strains. Meanwhile, this time can be efficiently utilized for conducting both preclinical and clinical studies to understand how antibody response changes to the COVID-19 vaccine, if there exist any, when they are given at a specific time of the day.

The circadian clock in CD8^+^ T cells plays an important role in response modulation that occurs during vaccination. In mice, this leads to the activation of higher numbers of T cells when vaccination with dendritic cells (loaded with ovalbumin peptides) is administered during the day compared to the night (Nobis et al., 2019). Attenuated encephalomyelitis vaccine when administered at 8:00 am was shown to reach the peak antibody titer 4 days earlier than those that were vaccinated at 8:00 pm (Feigin et al., 1967). Influenza vaccination administered in the morning between 9:00 and 11:00 am was shown to have a better antibody response against different influenza strains in humans compared to administration in the afternoon between 3:00 and 5:00 pm (Long et al., 2016). Timing thus seems to modulate antibody response and better outcomes are not always associated with morning administration. Hepatitis B vaccine, for instance, when administered between 1:00 and 3:00 pm resulted in significantly higher antibody response when compared with those who received the vaccine between 7:30 and 9:00 am (Pollman and Pollman, 1988). Additionally, antibody responses can be triggered better for protection against different viruses if the month of vaccine administration is strategically chosen in nations experiencing seasonal variations. For instance, B-cell maturation factor expression exhibits seasonal variation (Dopico et al., 2015), and these expressions are linked to responses conducive to trivalent influenza vaccine (Nakaya et al., 2011). Another study found a considerable association between the response of antibodies to rabies vaccine and vaccination month (Moore et al., 2006). Pathogen recognition receptors (PPR) also exhibit seasonal variability with an enhanced expression during winter, modulating vaccine response quality and providing enhanced defense against the virus causing yellow fever (Querec et al., 2009; Dopico et al., 2015). Overall, all these studies cumulatively suggest that timing might play a key role to further determine the efficacy of the COVID-19 vaccine. In these challenging times when the SARS-CoV-2 virus is posing a significant threat to vulnerable people, the findings of the above-mentioned studies on timed vaccine administration can be effectively leveraged to offer comprehensive protection against COVID-19.

## Conclusion

As the coronavirus pandemic continues to rage on and to counter its insidious effect, researchers are taking a closer look at sleep, an important contributor to optimal health. Throughout this pandemic, sleep has been negatively impacted due to anxiety induced by illnesses, financial insecurity, housing issues, or working as a frontline worker, social media news, lesser exposure to sunlight inside homes, and so on. Low-quality sleep has a disruptive effect on the immune system at the molecular level, greatly increasing the susceptibility to diseases. Proper sleep also makes recovery from sickness faster. Poor sleep also triggers heart ailments (e.g., blood pressure), kidney problems, diabetes, cancer, obesity, and hypertension. Amidst this pandemic, it is very essential to sleep well at night to render the immune system stronger. Quality sleep should complement nutritional food intake at proper times and physical activities. As daily routines have been thrown haywire, it is important to maintain a strict sleep schedule and wake up at the same time each day. Before going to sleep, relaxation-inducing activities like meditation, bath, book reading, or enjoying soul-soothing music can calm down the sympathetic nervous system, which helps facilitate sleep. Sleep interference can occur if we gaze at computers or tablets that do not have a blue light filter in place before bedtime, consume nicotine, alcohol, or caffeine, or take late-day naps. Unhealthy practices should be left behind, and conscious efforts need to be made to adopt a healthier lifestyle. Embracing healthier sleeping habits, as we have discussed in this review, will help strengthen and normalize the circadian rhythms naturally.

The outbreak of the new contagious virus, SARS-CoV-2, imposed unique challenges on the healthcare front, particularly in the selection of the appropriate therapeutics against the new infectious virus. Currently, there is still no specific treatment against the novel COVID-19. Since the beginning of the pandemic, there has been an urgent need for effective therapeutic agents against the SARS-CoV-2. To combat the COVID-19 pandemic, it is imperative that we collaboratively adopt novel interventions across various medical specialties. Here, we introduce a fundamental concept that is frequently discussed in sleep medicine, chronotherapy, and integrate it with the discussion of the several major classes of drugs that can be repurposed for the treatment and management of COVID-19. Accumulation evidence from the literature suggests that nearly half of all physiological functions, including the body’s pathogenic response, are controlled tightly by the circadian clock. Chronotherapy exploits this rhythmic pattern to its advantage to improve the outcome of different medical interventions as discussed throughout this review. This manuscript highlights that diurnal variation exists in the functioning of our immune system, *in vivo* cholesterol synthesis, coagulation, fibrinolysis and blood pressure, and that considering the time of drug administration might significantly improve the treatment and management of the novel coronavirus disease-19.

## Author Contributions

AG, AS, and IS conceived the ideas, collected the appropriate literature, drafted the outline, contributed to the writing of this review, updated the figures/schematics, and checked, edited, and approved the final version of the review. All authors contributed to the article and approved the submitted version.

## Conflict of Interest

The authors declare that the research was conducted in the absence of any commercial or financial relationships that could be construed as a potential conflict of interest.

## Footnotes

1 http://circadb.hogeneschlab.org/

## Supplementary Material

Supplementary Table 1 Drugs that may be repurposed for the treatment of SARS-CoV-2 and associated chronotherapy.
