# Supplementary material for: COVID-19: Sleep, Circadian Rhythms and Immunity – Repurposing Drugs and Chronotherapeutics for SARS-CoV-2
Source: Front Neurosci. 2021 Jun 18;15:674204. doi: 10.3389/fnins.2021.674204 (PMC8249936; doi:10.3389/fnins.2021.674204)
Supplement: Supplementary Table 1 — Drugs that may be repurposed for the treatment of SARS-CoV-2 and associated chronotherapy. [file Table_1.pdf]

Supplementary Table 1. Drugs that may be repurposed for the treatment of SARS-CoV-2 and associated chronotherapy.

Category	Drug Names / Classes	Evidence and scientific rationale for the treatment of SARS-CoV-2 infection and associated chronotherapeutic mechanisms	Citations
**Drugs that inhibit or block the life cycle of SARS-CoV-2**	**Antiviral (Remdesivir)**	• Effective against other coronaviruses such as MERS-CoV and SARS-CoV.<br>• Showed superior effect over placebo in shortening the recovery time of hospitalized COVID-19 patients.<br>***Evidence for chronotherapy***<br>• Host clock components regulate viral replication either directly or indirectly thereby modulate the viral load.<br>• In a retrospective study, CRP levels reduced significantly when the antiviral drug was administered in the morning compared to the evening.<br>• The dose of acyclovir needed to prevent HSV-2 infection during the active phase was four times more compared to the resting phase in mice.	(de Wit et al., 2020; Malin et al., 2020)<br>(Madsen, 2020)<br>(Edgar et al., 2016)<br>(De Giorgi et al., 2020)<br>(Matsuzawa et al., 2018)
**Drugs that counteract the insidious effects caused by SARS-CoV-2**	**Corticosteroids (Dexamethasone, Prednisone and Methylprednisolone)**	• Corticosteroids are anti-inflammatory drugs that suppress the activation of the immune system, thus prevents cytokine storm.<br>• Meta-analysis of clinical trials showed that corticosteroids reduced the risk of mortality and the duration of mechanical ventilation in patients suffering from ARDS and COVID-19.<br>***Evidence for chronotherapy***<br>• Elevated levels of pro-inflammatory cytokines throughout the nighttime.	(Channappanavar and Perlman, 2017)<br>(Mammen et al., 2020; Sterne et al., 2020)<br>(De Silva et al., 1984)

		• Prevents the nighttime rise in pro-inflammatory cytokines, particularly IL-6, which may be beneficial for COVID-19, as reported in studies from rheumatoid arthritis patients.	(De Silva et al., 1984; Buttgereit et al., 2008)
	**Blood thinners (Anticoagulants or antiplatelet drugs)**	• Increased rate of blood clots among hospitalized COVID-19 patients.<br><br>• Full dose anticoagulants when given to moderately ill hospitalized COVID-19 patients, the requirement for vital organ support such as ventilation and ICU was significantly reduced.<br><br>• Prophylactic anticoagulation administration did not increase the risk of serious bleeding in COVID-19 patients.<br><br>***Evidence for chronotherapy***<br><br>• Hypercoagulatory and hypofibrinolytic conditions are more frequent in the morning because of increased platelet activity and concentration of coagulation factors like Factor V, VII, and prothrombin fragment F_{1+2}, and D-dimer.<br><br>• Rivaroxaban and Aspirin both have been shown to exert better effects when taken in the evening compared to the morning.	(Malas et al., 2020)<br><br>(Health, 2021)<br><br>(Rentsch et al., 2021)<br><br>(Kapiotis et al., 1997)<br><br>(Bonten et al., 2015; Brunner-Ziegler et al., 2016; van Diemen et al., 2020)
	**Cationic Amphiphilic Drugs (Amiodarone, Chlorpromazine, and SERMs)**	• The anti-arrhythmic drug, amiodarone prevents the fusion of the viral envelop with the endosomal membrane and accumulates in late endosomes/ lysosomes, and disrupts the viral endocytic pathway.<br><br>• Amiodarone prevents entry of the Filovirus and methyldiethanolamine (metabolite) was able to inhibit Ebola virus entry.<br><br>• Amiodarone increased the survival of mice infected with the Ebola virus.	(Salata et al., 2015)<br><br>(Gehring et al., 2014; Salata et al., 2015)<br><br>(Madrid et al., 2015)

		• Amiodarone also inhibits other viruses like the Arenavirus, the SARS-CoV-1, and Hepatitis C virus.<br><br>• Arrhythmias are common among COVID-19 patients, and it is associated with higher morbidity and mortality.<br><br>• Chlorpromazine exhibits antiviral activity against the Crimean-congo hemorrhagic fever virus, Adenovirus, Ebola virus, MERS-CoV, and SARS-CoV.<br><br>• Chlorpromazine blocks the formation of clathrin-coated pits and thus prevents viral entry into the cells<br><br>• Selective estrogen receptor modulators (SERMs) have protective functions against MERS-CoV, Ebola virus, HSV-1, and HCV<br><br>• SERM, clomiphene possess antiviral activity against the Ebola virus by interfering with the late-stage fusion of the viral envelope and the endosomal membrane.<br><br>• Clomiphene blocks the Ebola virus entry by inhibiting the NPC1-dependent pathway, which has been hypothesized to increase cholesterol accumulation in the late endosomes and impair viral entry for SARS-CoV-2<br><br>***Evidence for chronotherapy***<br><br>• Prior studies suggest that cardiac arrhythmia peaks mostly between 6:00 am and 12:00 noon. Arrhythmogenesis appeared to be less frequent or suppressed during the nighttime.	(Stadler et al., 2008; Cheng et al., 2013; Gehring et al., 2014)<br><br>(Babapoor-Farrokhran et al., 2020)<br><br>(Bhattacharyya et al., 2010; Diaconu et al., 2010; Dyall et al., 2014; Ferraris et al., 2015)<br><br>(Daniel et al., 2015)<br><br>(Johansen et al., 2013; Murakami et al., 2013; Zheng et al., 2014)<br><br>(Nelson et al., 2016)<br><br>(Ghasemnejad-Berenji et al., 2020)<br><br>(Portaluppi and Hermida, 2007)

		• A relatively lower dose of chlorpromazine administered at 1:30 was able to show the same sedative effect than when administered at 7:30 (on a 24-hour clock).	(Nagayama et al., 1978)
	**Janus-associated Kinase Inhibitor (Baricitinib)**	• JAK inhibitors can prevent the phosphorylation of proteins that are involved in the signal transduction cascade of the Jak-Stat pathway and thereby reduce cytokine-mediated inflammation and collateral damage in the vital organs.<br><br>• Baricitinib significantly reduced the median number of days to recovery, from 18 to 10 days, in hospitalized COVID-19 patients requiring high-flow oxygen or non-invasive ventilation, when used together with remdesivir.<br><br>• The need for ventilation support or death was reduced by > 50% (34.9% to 16.9%) using Baricitinib compared to the placebo/control group.<br><br>• Baricitinib can successfully inhibit type-1 interferon response (exaggerated in COVID-19 patients) that increases ACE2 expression (in liver cells), the receptor that plays an essential role in the entry of SARS-CoV-2 in host cells to increase the viral load.<br><br>• Baricitinib is a potent inhibitor of the Numb-associated kinase (NAK) family of proteins, particularly AAK1, that plays a pivotal role in clathrin-mediated endocytosis, which further prevents viral entry into the cells.<br><br>***Evidence for chronotherapy***<br><br>• Studies have shown a peak in IL-6 levels during nighttime and early morning hours.	(Lin et al., 2020)<br><br>(Kalil et al., 2020)<br><br>(Stebbing et al., 2021).<br><br>(Stebbing et al., 2021)<br><br>(Conner and Schmid, 2002; Sorrell et al., 2016)<br><br>(Vgontzas et al., 2005)

		• A better outcome was observed when Baricitinib was administered during the time (evening) when the cytokine production was at the highest.	(Yaekura et al., 2020)
**Drugs that manage comorbidities and pleiotropic effects against SARS-CoV-2**	**Hyperlipidemia drug (Statins)**	• Anti-inflammatory effects can block the infectious potential of enveloped viruses (*in vitro*) and considerably reduce the mortality risk among COVID-19 patients.<br>• Independently associated with lower ICU admission among COVID-19 patients.<br>• Reduce hyperlipidemia and decrease cytokine levels with its pleiotropic effects under different non-infectious conditions.<br>***Evidence for chronotherapy***<br>• Cholesterol synthesis peaks between 12:00 am and 6:00 am.<br>• Administration of Statins in the evening is more effective than when taken in the morning.	(Rossi et al., 2020)<br>(Tan et al., 2020)<br>(Wassmann et al., 2003; Fang et al., 2005), NCT02056340<br>(Izquierdo-Palomares et al., 2016)<br>(Saito et al., 1991; Lund et al., 2002; Wallace et al., 2003; Ozaydin et al., 2006; Tharavanij et al., 2010).
	**Antihypertensive drugs (ACE inhibitors and ARBs)**	• Acute lung damage can be reduced by renin-angiotensin-aldosterone system inhibitors.<br>• Case fatalities are much higher in COVID-19 patients due to pulmonary hypertension. Hypertensive drugs are associated with decreased mortality.<br>***Evidence for chronotherapy***<br>• Blood pressure (BP) is higher during early mornings. Increased nighttime ambulatory BP is related to fatal and non-fatal cardiovascular events.	(Baral et al., 2020)<br>(Surveillances, 2020)<br>(Elliott, 1999)

		• Lessing BP at night and early morning hours markedly reduce cardiovascular events by administering hypertensive drugs before bedtime or in the evening.	(Hermida et al., 2007; Hermida and Ayala, 2009; Hermida et al., 2010; Hoshino et al., 2010; Zeng et al., 2011; Bowles et al., 2018; Hermida et al., 2020)
**Chronotherapeutic drugs that may be used against SARS-CoV-2**	**REV-ERBα agonists (e.g., SR9009 and GSK2667)**	• Boost the endurance performance by increasing mitochondria numbers/counts in skeletal muscles and significantly reduce cholesterol and body weight.<br>• Lower anxiety to a level as effective as benzodiazepine.<br>• Reduces inflammation by interfering with the production of inflammatory cytokines like TNFα, CCL2, and MMP-9 *in vitro* in nerve cells and *in vivo* rat lungs.<br>• Reducing blood vessel lesions and hardening of arteries.<br>• SR9009 can successfully inhibit positive-strand RNA viruses such as the Flaviviridae family (e.g., Hepatitis C virus, Dengue, and the Zika virus).<br>• Regulate HIV-1 replication by inhibiting promoter activity in $CD4^+$ T cells and macrophages.<br>• Successfully inhibits the replication of alphaviruses like Chikungunya and o'nyong'nyong virus by suppressing the synthesis of the structural proteins.<br>• Selectively regulate pro-inflammatory cytokines like IL-6, which has been considered as a prognostic marker for mortality among SARS-CoV-2 infected patients.	(Solt et al., 2012; Woldt et al., 2013)<br>(Banerjee et al., 2014)<br>(Li et al., 2014; Morioka et al., 2016)<br>(Sitaula et al., 2015)<br>(Zhuang et al., 2019)<br>(Borrmann et al., 2020)<br>(Hwang et al., 2018)<br>(Gibbs et al., 2012; Liu et al., 2020)
